# Functional overload attenuates plantaris atrophy in tumor-bearing rats

**DOI:** 10.1186/1471-2407-7-146

**Published:** 2007-08-02

**Authors:** Jeffrey S Otis, Simon J Lees, Jay H Williams

**Affiliations:** 1 Pulmonary, Allergy and Critical Care Medicine, Emory University School of Medicine and Atlanta VA Medical Center, Decatur, GA 30033, USA; 2 Department of Biomedical Sciences, University of Missouri-Columbia, Columbia, Missouri 65211, USA; 3 Department of Human Nutrition, Foods and Exercise, Virginia Polytechnic Institute and State University, Blacksburg, VA 24061, USA

## Abstract

**Background:**

Late stage cancer malignancies may result in severe skeletal muscle wasting, fatigue and reduced quality of life. Resistance training may attenuate these derangements in cancer patients, but how this hypertrophic response relates to normal muscle adaptations in healthy subjects is unknown. Here, we determined the effect of resistance training on muscle mass and myosin heavy chain (MHC) isoform composition in plantaris muscles from tumor-bearing (TB) rats.

**Methods:**

Age- and gender-matched Buffalo rats were used for all studies (n = 6/group). Suspensions of Morris Hepatoma MH7777 cells or normal saline were injected subcutaneously into the dorsum. Six weeks after cell implantation, muscles from TB rats were harvested, weighed and processed for ATP-independent proteasome activity assays. Once tumor-induced atrophy had been established, subgroups of TB rats underwent unilateral, functional overload (FO). Healthy, sham-operated rats served as controls. After six weeks, the extent of plantaris hypertrophy was calculated and MHC isoform compositions were determined by gel electrophoresis.

**Results:**

Six weeks of tumor growth reduced body mass and the relative masses of gastrocnemius, plantaris, tibialis anterior, extensor digitorum longus, and diaphragm muscles (p ≤ 0.05). Percent reductions in body mass had a strong, negative correlation to final tumor size (r = -0.78). ATP-independent proteasome activity was increased in plantaris muscles from TB rats (p ≤ 0.05). In healthy rats, functional overload (FO) increased plantaris mass ~44% compared to the contralateral control muscle, and increased the relative percentage of MHC type I and decreased the relative percentage of MHC type IIb compared to the sham-operated controls (p ≤ 0.05). Importantly, plantaris mass was increased ~24% in TB-FO rats and adaptations to MHC isoform composition were consistent with normal, resistance-trained muscles.

**Conclusion:**

Despite significant skeletal muscle derangements due to cancer, muscle retains the capacity to respond normally to hypertrophic stimuli. Specifically, when challenged with functional overload, plantaris muscles from TB rats displayed greater relative mass, increased percentages of MHC type I and decreased percentages of MHC type IIb. Therefore, resistance training paradigms should provide relative morphological and functional benefits to cancer patients suffering from muscle wasting.

## Background

Cachexia is a debilitating condition associated with several chronic disease states, including AIDS, cancer and congestive heart failure [1], and manifests in severe body wasting with specific insults to skeletal muscle. Cancer cachexia, also known as wasting syndrome, is a paraneoplastic syndrome that afflicts approximately two thirds of patients with advanced cancers and results in generalized weakness, asthenia, and a decreased ability to tolerate conventional therapies [2]. In fact, approximately 22–30% of all cancer deaths are directly attributable to complications arising from cachexia, primarily due to the deterioration of respiratory musculature and subsequent hypostatic pneumonia [3,4].

Invariably, the underlying cause of skeletal muscle wasting due to cancer cachexia is a severe metabolic disturbance as tumor and host contend for nutrients and energy substrates. While anorexia or reduced caloric intake may contribute to this wasting process, nutritional intervention strategies are often insufficient to prevent the development of cachexia [5], which reflects the complex pathology of the disease. Therefore, skeletal muscle wasting in cachectic cancer patients has been attributed to factors independent of nutritional status, including increased proinflammatory cytokines levels [2,6], increased levels of tumor-derived factors [7], or altered hormonal status [8]. Tumor-induced skeletal muscle protein loss appears to be due, in part, to increased activity of the ATP-ubiquitin-dependent proteasome system [9-11]. Using the aggressive Yoshida ascites hepatoma to produce rapid skeletal muscle atrophy, inhibition studies have suggested that other proteolytic systems, such as Ca^2+^-activated calpain proteases, lysosomal proteases or the ATP-independent-proteasome system, play only negligible roles in acute tumor-induced atrophy [9,10]. However, the regulation of skeletal muscle atrophy by the ATP-independent proteasome system during chronic tumor development remains poorly understood.

While the negative effects of cancer cachexia on skeletal muscle mass are well-established, few studies have attempted to identify the effects of cancer cachexia on properties that influence muscle function such as myosin heavy chain (MHC) isoform composition [12]. Certainly in other experimental models associated with skeletal muscle atrophy, increases in the relative distribution of fast MHC isoforms are evident [13-15]. In contrast, experimental models of resistance training (e.g., functional overload (FO) of the plantaris muscle) are associated with increased percentages of slower MHC isoforms [16]. While resistance training may attenuate muscle atrophy and improve muscle strength in cancer patients [17], its influence on MHC isoforms remains unresolved.

In these studies, we hypothesized that skeletal muscles from rats bearing a tumor derived from Morris hepatoma MH7777 cells would have increased ATP-independent proteasome activity, which may contribute to systemic skeletal muscle atrophy. We next tested the hypothesis that FO would prevent plantaris muscle atrophy and stimulate fast-to-slow MHC isoform transitions in tumor-bearing (TB) rats, both of which are characteristics of resistance-trained skeletal muscle [16]. Our data show that cancer cachexia does not affect the capacity of skeletal muscle to respond normally when challenged with a hypertrophic stimulus. These data also suggest that resistance training should provide relative morphological and functional benefits to cancer patients suffering from muscle wasting.

## Methods

### Animals

The Virginia Tech Animal Care Committee approved all experimental procedures. Female Buffalo rats (~150–175 g) were purchased from Harlan Sprague Dawley (Indianapolis, IN). All rats were housed in pairs under a 12:12 light-dark cycle at room temperature and provided free access to food (Harland Teklad 2018 rodent chow) and water. Rats were randomly assigned to one of four groups (n = 6/group): vehicle-injected, sham-operated (Veh); vehicle-injected, FO (FO); tumor-bearing, sham-operated (TB); and tumor-bearing, FO (TB-FO). Body, skeletal muscle and tumor masses were obtained at termination.

### Tumor model

The Morris hepatoma MH7777 cell line (ATCC #CRL-1601) was used to induce tumor formation and has previously been used to study derangements associated with cancer cachexia [18]. Cells were grown in Dulbecco's Modified Eagle's Medium (4.5 g glucose/L, L-glutamine, NaHCO_3_, and pyridoxine HCl) supplemented with 10% fetal bovine serum and 50 μg/ml gentamycin and maintained at 37°C and 5% CO_2_. Cells were trypsinized in a subconfluent state (~85–90% confluency), washed and resuspended in sterile Dulbecco's phosphate-buffered saline prior to injection. Rats in the TB groups received a suspension of cells (10^6 ^cells/300 μl) implanted subcutaneously on the dorsal side above the hip using a 22-G 1.5" needle. Pilot data indicated that this cell density and injection location produced average tumor masses of 6.36 ± 0.93 g after 4 weeks and significantly reduced skeletal muscle masses after 6 weeks. Functional overload surgeries did not affect tumor development. Total food intake/cage (data not shown) and common signs of distress (e.g., failure to groom) were monitored daily. Neither was altered in TB rats nor in rats that underwent FO surgeries. Control rats not receiving MH7777 cells were injected with 300 μl of sterile Dulbecco's PBS solution.

### Muscle preparation

Six weeks after MH7777 cell or vehicle injection, rats were anesthetized with intraperitoneal injections of Ketamine (80 mg/kg) and Xylazine (10 mg/kg). Soleus, gastrocnemius, plantaris, tibialis anterior, extensor digitorum longus and diaphragm muscles were removed, trimmed free of connective tissue and fat, blotted dry, weighed, frozen in isopentane cooled in liquid nitrogen and stored at -80°C until further analysis.

### ATP-independent proteasome activity assay

The ATP-independent proteasome activity assay measures fluorescence following chymotrypsin-like degradation of the fluorometric substrate, Suc-Leu-Leu-Val-Tyr-AMC (Bachem). This substrate has been used previously to analyze proteasome activity [19]. Briefly, fresh soleus and plantaris muscles were homogenized in ten volumes of buffer that contained: 1 M Tris (pH 7.6), 200 mM EDTA, and 0.1% β-mercaptoethanol. Samples were then centrifuged at 12,000 rpm for 20 minutes at 4°C. Supernatants were subjected to dialysis overnight at 4°C using Spectra/Por^® ^biotech membranes with a retention rating of 15,000 (Spectrum Laboratories, Inc.). The dialysis buffer contained: 20 mM Tris (pH 7.6), 20 mM NaCl, 1 mM MgCl2, 0.1 mM EDTA, 0.5 mM DTT, and 20% glycerol. Dialysate was then centrifuged at 15,200 g for 20 minutes at 4°C. A Perkin-Elmer luminescence spectrophotometer LS-50-B was set to an excitation wavelength of 380-nm and an emission wavelength of 460 nm. Reaction buffer containing: 1 M Tris (pH 8.0), 50 μM Suc-Leu-Leu-Val-Tyr-AMC, and 0.4% β-mercaptoethanol was warmed to 37°C prior to usage. Five-μl dialysate supernatant was added to 1 ml of reaction buffer in a glass cuvette. The reaction proceeded for 3 minutes and maximal ATP-independent proteasome activity (AU/min) was measured on the steepest part of the slope over a 20 second duration. Specific activities were adjusted for total protein content in each sample (AU/min/μg protein), as determined using the Bradford protein assay [20].

### Unilateral, functional overload surgeries

Unilateral, functional overload (FO) surgeries were performed 5 days prior to the introduction of MH7777 cells to avoid the inflammatory phase associated with this surgery. Unilateral FO surgery spares the non-operated contralateral leg to serve as an internal control, therefore allowing for precise measurements of hypertrophy [21].

Rats were anesthetized with a gas mixture of 2 L/min isofluorane and 3 L/min oxygen. Under aseptic conditions, a midline incision was made through the skin on the posterior aspect of the animal's lower leg on the same side as MH7777 cell implantation. The distal tendon of the gastrocnemius muscle was isolated from the tendons of its major synergists (soleus and plantaris) and transected. The distal portion of the gastrocnemius was removed, with particular care taken not to interrupt vasculature or innervation leading to the soleus or plantaris muscles. The skin was closed with 3-0 Ethilon suture. The incision was treated with a topical antibiotic (Bacitracin). Rats were returned to their cages and monitored daily for sudden changes in health or temperament.

### Electrophoretic separation of MHC isoforms

Frozen plantaris muscles were minced in 10 volumes of buffer containing: 20 mM Tris (pH 6.8), 250 mM sucrose, 5 mM EDTA, and 100 mM KCl. Plantaris samples were then homogenized by hand using glass tissue grinders kept on ice. Total protein was assayed according to the method of Bradford [20]. Protein samples were boiled for 2 minutes in buffer containing: 100 mM Tris-HCl (pH 6.8), 240 mM β-mercaptoethanol, 4.38 mM SDS, 3 μM bromophenol blue, and 20% glycerol to a final total protein concentration of 0.125 mg/ml. MHC isoforms were separated by sodium dodecyl sulfate-polyacrylamide gel electrophoresis (SDS-PAGE) as previously described [22]. Briefly, the stacking gel was composed of 30% glycerol, 4% acrylamide-N, N'-methylene-bis-acrylamide (bis) (50:1), 70 mM Tris (pH 6.7), 4 mM EDTA, and 0.4% SDS. The separating gel was composed of 30% glycerol, 8% acrylamide-bis (50:1), 200 mM Tris (pH 8.8), 100 mM glycine, and 4% SDS. Polymerization of the stacking and separating gels was initiated by adding 0.05% N, N, N', N'-tetramethylethylenediamine (TEMED) and 0.1% ammonium persulfate. One-μg of total protein was loaded per lane and separated in a Biorad mini-Protean 3 at a constant 70 volts supplied by a BioRad PowerPac 300 for 24 h. The mini-Protean 3 was stored at 4°C to minimize temperature fluctuations. The gels were stained with Coomasie Blue overnight at room temperature, destained with a solution containing 40% methanol and 10% acetic acid and analyzed with an Alpha Innotech IS-2000 video densitometric system. MHC isoform composition was expressed as percentages of the total MHC isoform pool.

### Statistics

One-way analyses of variance were performed for comparisons between control and TB rats. Two-way analyses of variance were performed for FO studies. These ANOVAs were followed by Student-Newman-Keuls post-hoc tests. All calculations were performed using SigmaStat v2.0 software. Significance was accepted at p ≤ 0.05.

## Results

### Tumor morphology, body and muscle masses

**Figure 1:**
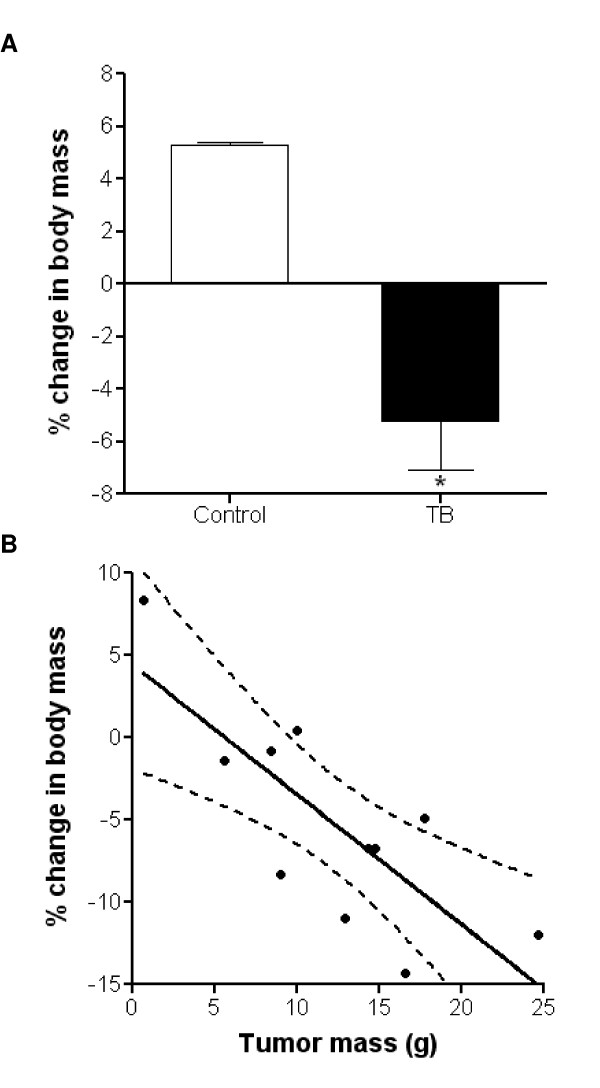
**Six weeks of tumor development induced body mass wasting**. (A) Tumors derived from Morris hepatoma MH7777 cells that were implanted on the dorsum of rats and allowed to develop for six weeks produced significant body mass reductions compared to healthy controls. (B) A strong, negative correlation existed between final tumor size and the percentage of body mass loss. Dashed lines represent the 95% confidence band. N = 6/group. *, Significantly different from control rats (p ≤ 0.05). TB, tumor-bearing.

Palpable tumors were evident ~16–19 days after MH7777 cell implantation. Final tumor masses after six weeks of development averaged 12.3 ± 1.8 g, which corresponded to ~10% of total carcass mass. TB rats lost an average of 5.21 ± 1.8% of their initial body mass (Figure 1A). In contrast, age-, gender- and weight-matched, healthy controls (Veh) gained an average of 5.3 ± 0.08% of body mass over these six weeks. Tumor size and percent change from initial body mass displayed a strong, negative correlation of r = -0.78 (Figure 1B).

**Figure 2:**
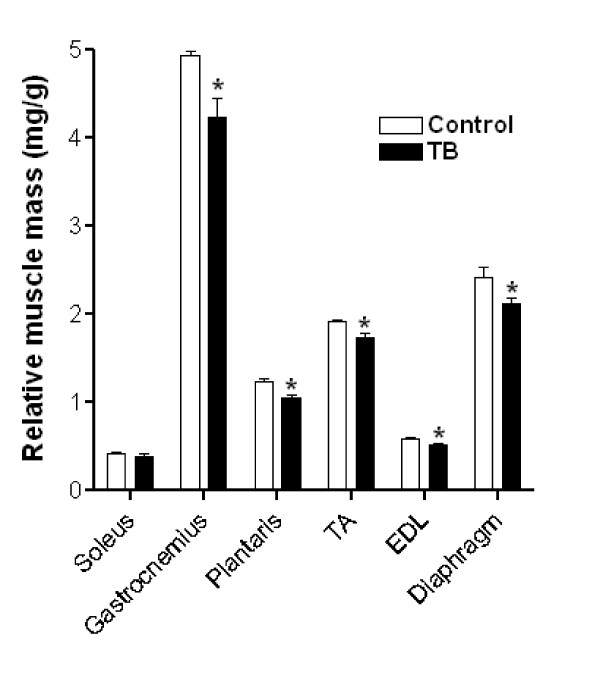
**Six weeks of tumor development induced skeletal muscle atrophy**. Several skeletal muscles, including gastrocnemius, plantaris, tibialis anterior, extensor digitorum longus and diaphragm, had reduced relative masses that could not be attributed to reduced caloric intake or anorexia. N = 6/group. *, Significantly different from control rats (p ≤ 0.05). TB, tumor-bearing.

Relative masses of several skeletal muscles were reduced, including gastrocnemius, plantaris, tibialis anterior, extensor digitorum longus and diaphragm (p ≤ 0.05). However, the soleus muscle was spared from tumor-induced atrophy (Figure 2).

### ATP-independent proteasome activity

**Figure 3:**
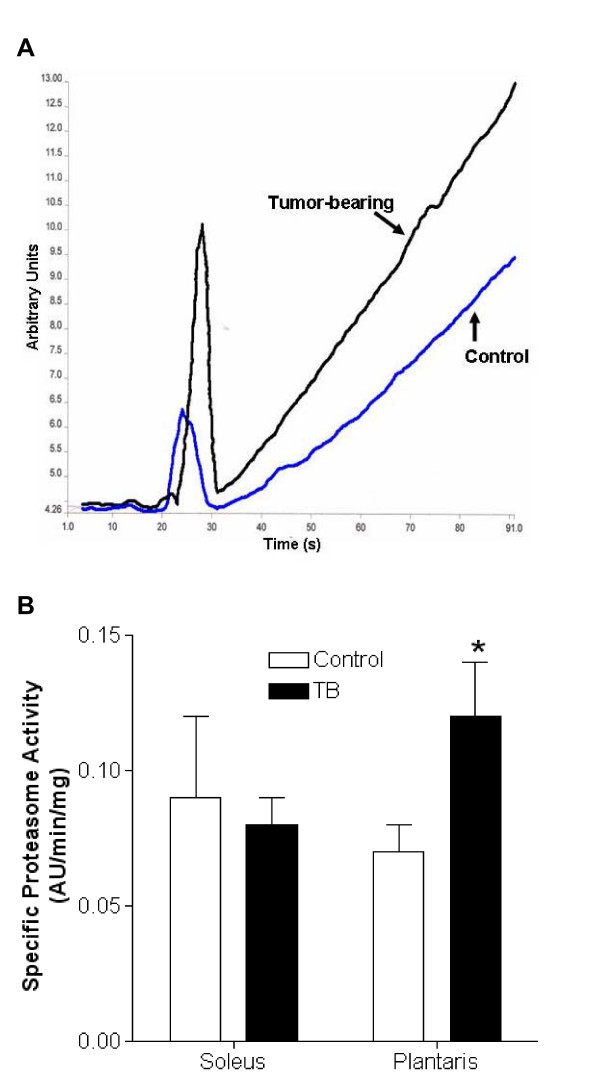
**ATP-independent proteasome activity is stimulated in plantaris muscles from tumor-bearing (TB) rats**. (A) Representative example of specific ATP-independent proteasome activity from plantaris muscles from TB and control rats. The sharp peaks between 22–30 seconds reflect the introduction of samples into the cuvette. Measurements of ATP-independent proteasome activity were made during the steepest part of the subsequent slope over a 20 second window. (B) Plantaris, but not soleus muscles, displayed increased relative proteasome activity due to the presence of the tumor. These data are expected considering that the soleus muscle was resistant to tumor-induced atrophy (Figure 2). N = 6/group. *, Significantly different from control rats (p ≤ 0.05).

Because little is known about the role of the ATP-independent proteasome component in tumor-induced skeletal muscle atrophy, we next determined the influence of this proteolytic system in soleus and plantaris muscles from TB rats. These two muscles were specifically chosen for proteolytic activity measurements because of their contrasting relative mass responses in TB rats (Figure 2). As expected, specific ATP-independent proteasome activity in soleus muscles from TB showed no difference compared to Veh (0.08 ± .01 vs. 0.09 ± 0.03 AU/min/μg, respectively) (Figure 3B). In contrast, atrophied plantaris muscles from TB rats had increased proteasome activity compared to Veh (0.12 ± 0.02 vs. 0.07 ± 0.01 AU/min/μg, respectively) (Figure 3A, B).

### Influence of functional overload on plantaris mass

**Figure 4:**
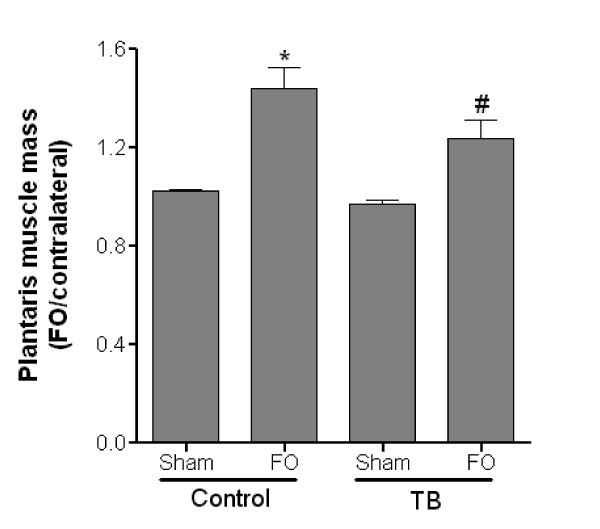
**Functional overload successfully prevents plantaris atrophy in TB rats**. Six weeks after functional overload surgeries were performed plantaris muscle masses were increased relative to contralateral control muscles, regardless of health status. N = 6/group. *, Significantly different from sham-operated control rats (p ≤ 0.05). #, Significantly different from sham-operated TB rats (p ≤ 0.05). FO, functional overload; TB, tumor-bearing.

Because plantaris muscles were atrophied in TB rats, we next attempted to attenuate these derangements by performing functional overload (FO) surgeries to stimulate growth. In healthy control rats, FO resulted in ~44% increase in mass relative to the contralateral plantaris (Figure 4) (p ≤ 0.05). Likewise, FO in TB rats resulted in ~24% increase in plantaris mass (p ≤ 0.05). Interestingly, there was no difference in the hypertrophic response in plantaris muscles from FO or TB-FO groups suggesting that the intrinsic and extrinsic signaling mechanisms that stimulate muscle hypertrophy are not affected by the atrophic signals caused by the tumor (Figure 2).

### Influence of functional overload on MHC isoform composition

**Figure 5:**
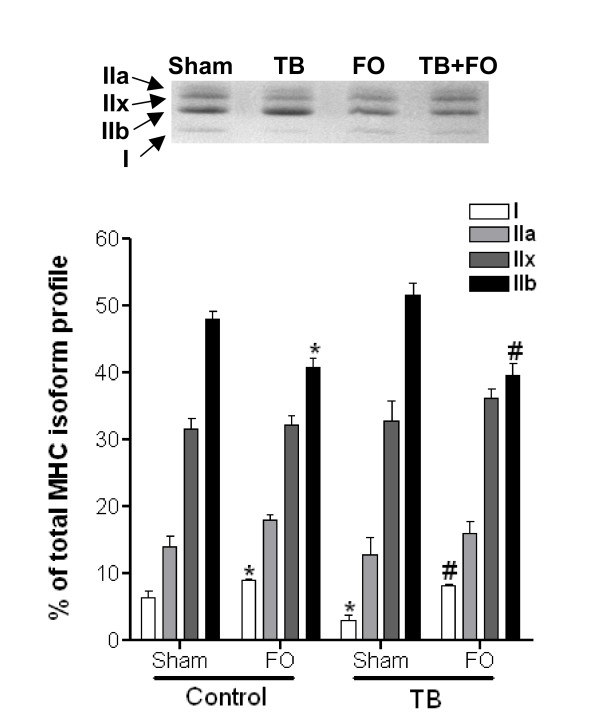
**Functional overload normalizes myosin heavy chain isoform profiles in TB rats**. Plantaris muscles from TB rats had decreased relative percentages of slow, MHC type I isoform which is suggestive of a faster MHC isoform profile. Similar to healthy rats, six weeks after functional overload surgeries were performed plantaris muscle from TB rats had increased relative percentages of slow, MHC type I isoform and decreased relative percentages of fast, MHC type IIb isoform. In fact, the MHC isoform profiles were similar in plantaris muscles from control or TB rats following FO surgeries. N = 6/group. *, Significantly different from sham-operated control rats (p ≤ 0.05). #, Significantly different from sham-operated TB rats (p ≤ 0.05). FO, functional overload; TB, tumor-bearing.

In healthy rats, plantaris muscles subjected to FO had increased relative percentages of slow, MHC type I isoform (9.0 ± 0.3% vs. 6.4 ± 0.8%, respectively) and decreased relative percentages of fast, MHC type IIb (40.8 ± 1.4% vs. 47.9 ± 1.3%, respectively) compared to sham-operated controls (Figure 5) (p ≤ 0.05). In sham-operated TB rats, plantaris muscles had decreased relative percentages of MHC type I compared to muscles from sham-operated healthy controls (2.9 ± 0.9% vs. 6.4 ± 0.8%, respectively) (p ≤ 0.05). However, plantaris muscles from TB-FO rats displayed increased relative percentages of MHC type I (8.2 ± 0.3% vs. 2.9 ± 0.9%, respectively) and decreased relative percentages of MHC type IIb (39.7 ± 1.6% vs. 51.6 ± 1.8%, respectively) compared plantaris muscles from sham-operated TB rats (p ≤ 0.05). Similar to the hypertrophic responses due to FO (Figure 4), no difference was detected in the relative MHC isoform profiles in plantaris muscles from FO or TB-FO rats.

## Discussion

We showed that tumor-bearing (TB) rats had significantly reduced body and skeletal muscle masses. Atrophied skeletal muscles from TB rats were also associated with increased ATP-independent proteasome activity. Importantly, the major finding of this study was that, despite the underlying atrophic effects produced by the tumor, skeletal muscle retains its ability to adapt to increased loading, including hypertrophy and establishment of a slower MHC isoform profile.

### Influence of tumor on body and skeletal muscle masses

Rats that developed solid tumors six weeks after subcutaneous implantation of Morris hepatoma MH7777 cells had ~5% decrease in body mass relative to initial body mass. This wasting was due, in part, to atrophy of several skeletal muscles, including gastrocnemius, plantaris, extensor digitorum longus, tibialis anterior and diaphragm. Importantly, the presence of the tumor did not appear to alter food intake nor did the location of the tumor on the dorsum restrict normal muscle growth (e.g., crush injury). Therefore, the decrease in body and skeletal muscle masses were likely due to systemic responses. Studies using other models of cancer cachexia-producing cell lines, including Yoshida AH-130 rat ascites hepatoma or C26 adenocarcinoma cell lines, despite having different tumor incubation periods, have reported similar magnitudes of wasting [9,12]. Together, these reports support the notion that cancer cachexia may produce general systemic atrophy. Interestingly, the soleus muscle appeared to be spared from tumor-induced atrophy. Because the soleus is a principle postural muscle in the hindlimb of rats, the location of the tumor on the dorsum of the rats and the extra mass it forced the animal to support may have provided a significant stimulus to prevent atrophy. This explanation seems reasonable when one considers that final tumor masses averaged 12.3 g, which corresponded to ~10% of total body weight. Alternatively, a longer duration of tumor growth may have been required to produce significant soleus atrophy. Interestingly, mice injected with murine C26 adenocarcinoma cells experienced significant soleus atrophy [12]. This apparent discrepancy with the current study may be due, in part, to the differing MHC isoform compositions between rat and mouse soleus muscles. The comparatively slower MHC phenotype of the rat soleus may have provided some measure of protection against tumor-induced atrophy.

Tumor-induced muscle atrophy appeared to be due, in part, to increased proteasome activity. Previous studies have demonstrated that the ATP-dependent-ubiquitin-proteasome pathway plays a central role in regulating muscle atrophy associated with cancer cachexia [9,23]. This pathway may be stimulated by a variety of tumor-induced agents, such as glucocorticoids, inflammatory cytokines, proteolysis-inducing factor (PIF), or oxidative stress [23]. Here, we showed that ATP-independent proteasome activities were stimulated in plantaris muscles from TB rats. Not surprisingly, there was no difference in proteasome activity in soleus muscles from TB rats, which did not experience tumor-induced atrophy. While metalloprotease or serine proteases may be responsible for ATP-independent proteasome activity [24], their target substrates are still largely undefined. However, myoglobin, the primary oxygen-carrying protein primarily expressed in oxidative skeletal muscle, appears to be primarily degraded by ATP-independent proteolytic mechanisms [24]. Interestingly, we show that plantaris muscles from TB rats may be becoming less oxidative, based on MHC isoform expression profiles. Certainly, adaptation to MHC isoform composition and metabolism may occur independently [14], but one possible explanation for this transition toward a more fatigable profile may be due, in part, to ATP-independent degradation of myoglobin.

### Influence of FO interventions on plantaris mass

Several laboratories have reported ~30–100% increases in plantaris muscle mass following various durations of compensatory hypertrophy [25]. In our healthy rats, six weeks after FO surgeries, plantaris muscles were ~44% larger than contralateral controls. Importantly, FO plantaris muscles from TB rats increased in relative mass by ~24% compared to muscles from sham-operated TB rats. In concert with our results, electrical stimulation of extensor digitorum longus muscles from tumor-bearing mice attenuated muscle wasting and protein depletion [26], which suggests that the normal hypertrophic signaling mechanisms and the capacity of skeletal muscle to respond to these signals remains intact. Yet, gene expression of insulin-like growth factor-1 (IGF-1), a potent anabolic hormone, was down-regulated in skeletal muscles from tumor bearing rats [8]. Indeed, more work is required to clarify the effects of resistance training on hypertrophic signal mechanisms in cancer patients.

### Influence of FO interventions on MHC isoform composition

Myosin heavy chain isoform composition is regulated, in part, by the amount and pattern of electrical activity a muscle receives [27]. For example, reduced neuromuscular activity due to spinal cord transection [14] or spinal cord isolation [28] increases the percentage of fast fiber-like protein phenotypes. In contrast, increased neuromuscular activation due to tonic, low frequency stimulation [29] or FO [16] induces the slow fiber-like protein phenotype. Importantly, tumors were not located near motor neurons that innervate the plantaris. Therefore, the conductance of electrical activity from the spinal cord to the plantaris was likely unaltered. This is supported by the observation that TB rats appeared pain-free and ambulated normally.

Myosin heavy chain isoform expression patterns have also been linked to mechanisms independent of neuromuscular activity. For example, increased plasma TNF-α levels have been shown to induce a faster MHC isoform profile in tibialis anterior muscles from rats with congestive heart failure [30]. Interestingly, levels of TNF-α are increased in cachectic cancer patients [31,32] and are elevated in severe combined immunodeficient (SCID) mice implanted with Morris hepatoma MH7777 cells [33]. Therefore, tumor-induced cytokines, such as TNF-α, may be responsible for the faster MHC isoform profile evident in plantaris muscles from TB rats. Because these cytokines are also implicated in stimulating proteasome activity [23], the susceptibility of specific isoforms to proteasome-mediated degradation may influence the overall MHC profile. However, neither translational nor post-translational protein modifications appear to be responsible for the slow-to-fast MHC isoform transition that occurs in TB animals [12]. Interestingly, plantaris muscles from mice bearing a murine C-26 adenocarcinoma showed no change to MHC isoform composition. This apparent discrepancy with the current study might be due to the different animal models used (mouse vs. rat), the different cell lines used to induce tumor formation (murine C-26 adenocarcinoma vs. MH7777 hepatoma cells), duration of tumor development, and/or the level of physical activity of the animals.

Importantly, plantaris muscles from TB rats retain the capacity to establish a slow MHC fiber profile comparable to that of resistance-trained muscle from healthy animals. The establishment of a slower MHC isoform profile may have significant physiological consequences, such as increased resistance to fatigue, improved oxidative capacities, and exercise tolerance [34,35]. In muscles from healthy animals, transitions toward a slower MHC isoform profile have been attributed to a variety of factors that are sensitive to increased neuromuscular activity, such as the Ca^2+^-dependent phosphatase calcineurin [36] or peroxisome-proliferator-activated receptor-γ co-activator-1 (PGC-1α) [37]. Recently, skeletal muscle atrophy due to cancer cachexia has been linked to reduced PGC-1α gene expression [38]. Because PGC-1α expression is stimulated by exercise [38,39], this transcription factor may provide an intriguing target to ultimately reduce the skeletal muscle derangements associated cancer cachexia.

## Conclusion

We have demonstrated that the decline in muscle mass in TB rats may be due, in part, to increased ATP-independent proteasome activity. Further, tumor-induced atrophy can be attenuated by an experimental model of resistance training. Resistance training may also normalize tumor-induced alterations to MHC isoform composition. In fact, the morphological gains achieved from FO in the plantaris muscle from TB rats were comparable to those from healthy controls. These data suggest that resistance training may provide relative benefits to cancer patients, including decreased weakness due to atrophy, improved tolerance to conventional therapies, and a better quality of life.

## Abbreviations

AIDS, acquired immunodeficiency syndrome; ATP, adenosine triphosphate; FO, functional overload; IGF-1, insulin-like growth factor-1; MHC, myosin heavy chain; PGC-1α, peroxisome-proliferator-activated receptor-γ co-activator-1; PIF, proteolysis-inducing factor; SCID, severe combined immunodeficiency disease; TB, tumor-bearing; TNF-α, tumor necrosis factor-α; Veh, vehicle

## Competing interests

The author(s) declare that they have no competing interests.

## Authors' contributions

JSO conception and design, data collection and analysis, figure and manuscript preparation. SJL assistance with FO surgeries and Morris hepatoma MH7777 cell implantations, editorial support and contributing important intellectual content. JHW concept, design and editorial support, research fund collection.

## Pre-publication history

The pre-publication history for this paper can be accessed here:


